# Association of mean platelet volume with incident type 2 diabetes mellitus risk: the Dongfeng–Tongji cohort study

**DOI:** 10.1186/s13098-018-0333-6

**Published:** 2018-04-10

**Authors:** Zhaoyang Li, Jing Wang, Xu Han, Jing Yuan, Huan Guo, Xiaomin Zhang, Dan Zheng, Yuhan Tang, Handong Yang, Meian He

**Affiliations:** 1 Department of Occupational and Environmental Health and State Key Laboratory of Environmental Health for Incubating, School of Public Health, Tongji Medical College, Huazhong University of Science and Technology, 13 Hangkong Rd, Wuhan, 430030 Hubei China; 2 Dongfeng Central Hospital, Dongfeng Motor Corporation and Hubei University of Medicine, Shiyan, Hubei China

**Keywords:** Type 2 diabetes mellitus, Means platelet volume, Cohort study

## Abstract

**Background:**

Most of prior studies to demonstrate the association between mean platelet volume (MPV) and type 2 diabetes mellitus (T2DM) risk were cross-sectional design with inconsistent results. In the present prospective cohort study, we aimed to explore the relationship between MPV and incident T2DM risk among a middle-aged and older Chinese population.

**Methods:**

This prospective study included 14,009 individuals derived from the Dongfeng–Tongji cohort which was launched in 2008. A total of 997 incident T2DM patients were diagnosed during the mean 4.51 years of follow-up period. MPV levels were divided into quartiles. The adjusted hazard ratios (HRs) and 95% confidence intervals (95% CIs) of incident T2DM was estimated by Cox proportional hazard models.

**Results:**

Compared with study participants with MPV < 7.49 fL, the HRs of T2DM incidence were 1.39 (95% CI 1.11–1.75), 1.14 (0.90–1.44), and 1.39 (95% CI 1.07–1.81) in study participants with 7.49 ≤ MPV < 8.43 fL, 8.43 fL ≤ MPV < 9.69 fL and MPV ≥ 9.69 fL, respectively. This positive association was more pronounced after exclusion of the newly diagnosed incident cases during the first 2 years follow-up. Further adjustment for baseline fasting blood glucose level (FBG) did not materially alter the positive association. The positive association was particularly evident among females, non-current smokers and study participants with FBG level less than 5.6 mmol/L at baseline.

**Conclusion:**

Higher levels of MPV were independently associated with increased incident risk of T2DM in a middle-aged and older Chinese population.

**Electronic supplementary material:**

The online version of this article (10.1186/s13098-018-0333-6) contains supplementary material, which is available to authorized users.

## Background

Diabetes mellitus (DM) is a major problem worldwide and the prevalence increased rapidly especially in developing countries [1]. In China it is estimated that DM prevalence increased to 11.6% (113.9 million adults) [2]. Studies indicated that inflammation played an important role in the development of DM [3]. Systemic sub-clinical inflammation has been implicated in the development of type 2 diabetes (T2DM) [4]. Inflammatory biomarkers, such as white cell counts (WBC), C-reactive protein (CRP), tumor necrosis factor-α (TNF-α), and interleukin-6 (IL-6), were showed to be correlated with prevalent and incident diabetes [5, 6].

Platelets, the second most abundant cell type in the circulation, play a classical role in thrombosis and homeostasis [7]. In recent decades, an increasing number of evidence showed that platelets were also related to inflammation [8]. Mean platelet volume (MPV), an important morphological parameters of platelets and an easily accessible indice in routine blood test, could reflect the size and activity of platelet [9]. Higher MPV level indicates larger platelets, which are metabolically and enzymatically more active [10]. In recent decade, MPV was defined as an inflammatory marker in different diseases such as fever and inflammatory bowel disease [11, 12].

Studies showed that DM cases have increased platelet activity [13]. A latest meta-analysis [14] which included 30 case–control and cross-sectional studies found that MPV was significantly higher in T2DM cases than study participants without DM. Nevertheless, other cross-sectional studies [15, 16] and a cohort study [17] exploring the relationship between MPV and diabetes obtained inconsistent findings. In the National Health and Nutrition Examination Survey the researchers reported that MPV was strongly and independently associated with the presence and severity of diabetes in study participants with diabetes [15]. However, the large prospective study to evaluate the impact of diabetes on MPV found no associations between DM and MPV [17].

Most published studies aimed to investigate the relationship between MPV and the prevalence and severity of diabetes [15] and its complications [18, 19]. However, few study examined causal effect of MPV and incident risk of T2DM. In addition, most of the previous studies were conducted in Europeans and the sample size was relatively small. Therefore, it still remains to elucidate whether MPV level is associated with incident diabetes risk, especially in other populations including the Chinese population.

In the current study, we aimed to investigate whether MPV is independently associated with the risk of incident T2DM in a large cohort of middle-aged and older Chinese adults. We further investigated whether the association could be modified by different characteristics or health status.

## Methods

### Study population

The study design, methods and other detailed information of the Dongfeng–Tongji (DFTJ) cohort have been described elsewhere [20]. Briefly, a total of 27,009 retired employees of the Dongfeng Motor Corporation (DMC) were recruited in the cohort and completed baseline questionnaires, medical examinations, and provided baseline blood samples between September 2008 and June 2010, and were followed until October 2013. At the first follow-up survey in 2013, participants repeated the questionnaire interview, physical examinations, and blood collection. In total, 25,978 individuals (96.2% of those at baseline) completed the first follow-up until October 2013.

Of these 27,009 individuals, we excluded participants with myocardial infarction (MI), coronary heart diseases (CHD), stroke, tumor (n = 5258), DM (n = 3080), and who was using anti-coagulation drugs, aspirin and thrombolytic drugs (n = 2182), with abnormal platelet count (PLT) data (n = 13) as well as those with missing data on baseline MPV (n = 2467) resulting in a final study sample of 14,009 participants (5980 males and 8029 females with a mean age of 62.23 years). This study was approved by the Ethics and Human Subject committee of Tongji Medical College, Dongfeng General Hospital, and DMC. All study participants provided informed consents.

### Assessment of covariates

Trained interviewers administrated interview questionnaire face to face to collect baseline information including socio-demographic characteristics (age, sex, and education), lifestyle habits such as smoking status (current, former, never), alcohol drinking status (current, former, never), physical activity, occupational history, environmental exposures, and family and medical histories. Participants who smoke at least one cigarette per day for more than half a year were defined as current smokers. Those who drink alcohol at least one time per week for more than half a year were defined as current alcohol drinkers. Physical activity was considered as those who regularly exercised at least 20 min per time for more than 6 months. Physical examination was performed at DMC-owned hospitals by trained physicians, nurses and technicians. Standing height, body weight and waist circumference were measured in individuals with light indoor clothing and without shoes. B-scan ultrasonography of the liver, gall bladder, spleen, kidney, prostate (for males) and uterus, ovaries and fallopian tubes (for females) were conducted. Body mass index (BMI) was calculated as weight (kilogram) divided by height (meter) squared. Hypertension was defined as individuals with self-reported physician diagnosed hypertension, or blood pressure ≥ 140/90 mmHg, or current use of antihypertensive medication. Hyperlipidemia was defined as total cholesterol > 5.72 mmol/L or triglycerides > 1.70 mmol/L at medical examination, or a previous physician diagnosis of hyperlipidemia, or taking lipid lowering medication.

Blood samples were collected after an overnight fast. Platelet counts (PLT), MPV, white blood counts (WBC), total cholesterol, triglycerides, high-density lipoprotein cholesterol (HDL-C), low-density lipoprotein cholesterol (LDL-C), fasting blood glucose (FBG), and hemoglobin A1c (HbA1c) were determined at the DMC-owned hospital’s laboratory. MPV, PLT and WBC were measured using a fully automated analyzer CELL-DYN 3700 (Abbott Laboratories. Abbott Park, Illinois, USA). The level of FBG was determined by aeroset automatic analyzer (Abbott Laboratories. Abbott Park, Illinois, USA) and the HbA1c level was measured with high-performance liquid chromatography D-10 system (Bio-Rad Laboratories. Hercules, CA, USA). The serum lipids were measured with the architect ci8200 automatic analyzer (Abbott Laboratories. Abbott Park, Illinois, USA).

### Ascertainment of baseline and incident diabetes

The diagnosis of DM was based on the American Diabetes Association (ADA) criteria [21] when meeting any of the following criteria: (1) self-reported of physician’s diagnosis of diabetes, (2) FBG level ≥ 7.0 mmol/L, (3) HbA1c level ≥ 6.5%, (4) usage of diabetes medication (insulin or oral hypoglycemic agent). The patients were those occurred after baseline survey but before the end of October 2013. A total of 997 incident patients were diagnosed during the follow-up period.

### Statistical analysis

The baseline continuous variables are expressed in means (SD) and compared by Student’s t test or analysis of variation (ANOVA) unless otherwise specified. The Categorical variables are presented as numbers and percentages and compared by Chi square analysis or Fisher’s exact test. A two-sided P value of less than 0.05 was considered to indicate statistical significance.

Eligible participants were grouped according to quartiles of MPV, and the first quartile (Q1) was regarded as the reference group. Cox proportional hazards regression model was used to evaluate the relationship between MPV and incident DM risk adjusting for covariates including age, sex, smoking status, alcohol drinking status, education, physical activity (hours per week), BMI, examination center, hypertension, hyperlipidemia, family history of DM, WBC, and PLT.

Among women, number of children, menopausal status, hormone replacement therapy, and contraception status were additionally adjusted in the Cox regression models.

Stratified analyses were further performed by baseline characteristics (including age [< 60, ≥ 60 years], sex, BMI [< 25, ≥ 25 kg/m^2^], current smoking [yes, no], current drinking [yes, no], hypertension [yes, no], hyperlipidemia [yes, no], WBC [< 5.7E9/L, ≥ 5.7E9/L], and baseline FBG [< 5.6, ≥ 5.6 mmol/L]). Moreover, to analyze potential interactions between MPV and main characteristics, an interaction product term was included in the model.

Finally, in order to eliminate the reverse causality between MPV and DM, we conducted sensitivity analysis by exclusion of the incident diabetes patients diagnosed during the first 2 years of follow-up. To examine whether the potential association between MPV and DM risk was due to the FBG levels at baseline, we further adjusted for baseline FBG concentrations in the full adjustment model.

## Results

### Baseline characteristics

Baseline characteristics of the participants according to the quartile of MPV are summarized in Table 1.

**Table 1:** Baseline characteristics of the study population (n = 14,009)

Variables	MPV (fL)				*P* value
	Q1 < 7.49	Q2 7.49–8.43	Q3 8.43–9.69	Q4 ≥ 9.69	
Participants	3513	3493	3502	3501	
Males, %	44.9	45.2	41.6	39.0	< 0.001
Age, years	62.35 (7.58)	62.44 (7.84)	62.26 (7.79)	61.88 (7.85)	0.015
Education, %					0.096
Primary or below	28.5	29.1	26.2	29.8	
Junior high school	36.7	35.8	37.6	35.8	
High school	25.8	25.1	25.4	23.9	
College or above	8.9	10.0	9.9	9.7	
Body mass index, kg/m^2^	24.24 (3.48)	24.11 (3.31)	24.06 (3.25)	24.93 (3.21)	0.097
Current smoking (yes, %)	20.0	20.7	18.8	16.7	< 0.001
Current drinking (yes, %)	23.9	23.6	22.7	21.0	0.015
Physical activity (yes, %)	88.2	87.6	89.3	89.9	0.007
Family history of diabetes (yes, %)	4.1	4.0	4.1	3.3	0.173
Hypertension (yes, %)	26.2	23.9	24.5	24.4	0.270
Hyperlipidemia (yes, %)	12.9	14.1	14.4	12.9	0.220
Incident diabetes (yes, %)	6.6	6.9	7.8	7.2	0.063
Systolic blood pressure (mmHg)	127.91 (18.39)	127.42 (18.39)	126.70 (18.41)	127.19 (18.69)	0.054
Diastolic blood pressure (mmHg)	78.61 (10.84)	77.49 (10.71)	76.51 (10.81)	77.17 (10.84)	< 0.001
Total cholesterol (mmol/L)	5.19 (0.88)	5.17 (0.94)	5.17 (0.93)	5.12 (0.95)	0.031
Triglycerides (mmol/L)	1.34 (0.86)	1.33 (0.92)	1.34 (0.97)	1.37 (0.87)	0.369
HDL-cholesterol (mmol/L)	1.44 (0.38)	1.39 (0.36)	1.47 (0.40)	1.54 (0.45)	< 0.001
LDL-cholesterol (mmol/L)	3.34 (0.79)	3.09 (0.81)	3.01 (0.92)	2.92 (0.77)	< 0.001
Fasting blood glucose (mmol/L)	5.53 (0.55)	5.54 (0.54)	5.54 (0.59)	5.44 (0.63)	< 0.001
Mean platelet volume, fL	6.77 (0.54)	7.95 (0.28)	9.01 (0.36)	11.16 (1.49)	< 0.001
Platelet counts, 10E9/L	200.00 (56.28)	196.92 (52.89)	190.00 (53.30)	173.06 (52.22)	< 0.001
White blood cell count, 10E9/L	5.70 (1.91)	5.84 (1.53)	6.00 (1.54)	6.01 (1.60)	< 0.001

Continuous variables were presented as mean (SD). Dichotomous variables were presented as n (%)

During 60,761.74 person-years of follow up, we identified a total of 997 incident diabetes cases. Among the 14,009 study participants, 42.7% were men and mean age was 62.23 years old. Overall, there were 25.07, 24.93, 24.99 and 24.99% of participant with MPV level < 7.49 fL, 7.49 ≤ MPV < 8.43 fL and 8.43 fL ≤ MPV < 9.69 fL and MPV ≥ 9.69 fL, respectively. Compared with Q1, individuals with higher levels of MPV were more likely to be younger, females, with higher levels of triglycerides, HDL-cholesterol, and WBC, and with lower levels of total cholesterol, LDL-cholesterol, and PLT. Also, study participants with higher levels of MPV were less likely to be current smokers and current drinkers. The baseline characteristics according to the quartile of MPV in women and men respectively were presented in Additional file 1: Table S1.

### Hazard ratios (HRs) of DM according to quartiles of MPV

As shown in Table 2, compared with study participants in Q1, the HRs and 95% CI of incident DM for individuals in Q2, Q3, and Q4 were 1.39 (1.11–1.75, *P *= 0.005), 1.14 (0.90–1.44, *P *= 0.277) and 1.39 (1.07–1.81, *P *= 0.014) respectively after adjustment for potential confounders. To reduce the possibility of reverse causality between MPV and DM events, sensitivity analyses were conducted by exclusion of the incident DM diagnosed during the first 2 years of follow-up (2009 and 2010, n = 249). After exclusion the positive association were even stronger (Q2 vs. Q1 HR: 1.44; 95% CI 1.13–1.84 and Q4 vs. Q1, HR: 1.51; 95% CI 1.14–1.99, *P* for trend = 0.028). In the full adjustment model we further adjusted the baseline FBG levels and the positive association did not alter materially (Q2 vs. Q1 HR: 1.39; 95% CI 1.10–1.74 and Q4 vs. Q1, HR: 1.33; 95% CI 1.02–1.73).

**Table 2:** Multivariate adjusted hazard ratios with 95% CI for T2DM incidence

	MPV (fL)				*P* trend*
	Q1	Q2	Q3	Q4	
MPV	< 7.49	7.49–8.43	8.43–9.69	≥ 9.69	
Median of MPV	6.90	7.95	8.99	10.7	
Cases, n (person-years)	231 (15,301.77)	242 (15,059.60)	272 (15,194.28)	252 (15,206.09)	
Model 1	Ref	1.24 (1.04, 1.49)	1.02 (0.88, 1.22)	1.17 (0.98, 1.39)	0.320
Model 2	Ref	1.37 (1.08, 1.63)	1.07 (0.86, 1.32)	1.25 (0.99, 1.58)	0.252
Model 3	Ref	1.39 (1.11, 1.75)	1.14 (0.90, 1.44)	1.39 (1.07, 1.81)	0.085
Sensitivity analysis					
Excluding participants who developed diabetes during the first 2 years of follow-up^a^	Ref	1.44 (1.13, 1.84)	1.16 (0.90, 1.49)	1.51 (1.14, 1.99)	0.028
Further adjustment for FBG at baseline	Ref	1.39 (1.10, 1.74)	1.10 (0.87, 1.39)	1.33 (1.02, 1.73)	0.193

Model 1: adjusted for age, sex

Model 2: additionally adjusted for BMI, smoking status, drinking status, education, physical activity, hypertension, hyperlipidemia, family history of DM, and examination center

Model 3: additionally adjusted for WBC and PLT

^a^ Sensitivity analysis was performed by excluding the newly diagnosed incident diabetes patients during the first 2 years of follow-up (n = 249)

* *P* value when assigning the median value to each quartile and entered as a continuous variable in the models

### Stratification and interaction analysis

**Fig. 1:**
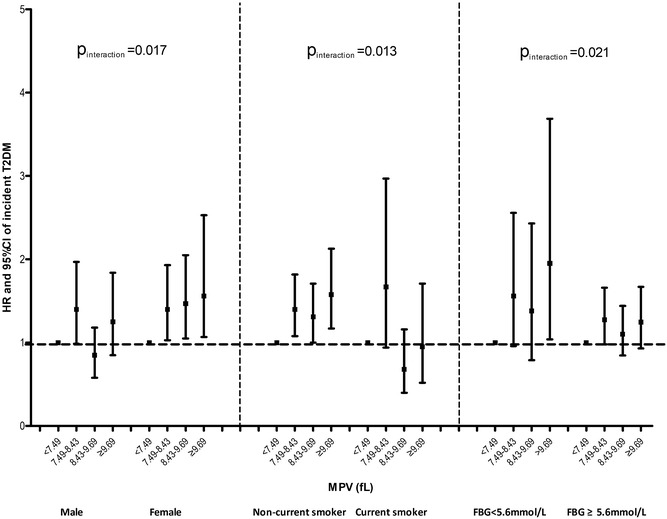
Adjusted HR (95% CI) for incident T2DM in individuals with higher MPV levels compared with the reference group (MPV < 7.49 fL). All covariates were age, sex, BMI, smoking status, drinking status, education, physical activity, hypertension, hyperlipidemia, family history of DM, examination center, WBC and PLT. Each group adjusted for the other covariates except itself

Stratified analyses were further performed by stratifying the baseline characteristics (including age [< 60, ≥ 60 years], sex, BMI [< 25, ≥ 25 kg/m^2^], current smoking [yes, no], current drinking [yes, no], hypertension [yes, no], hyperlipidemia [yes, no], WBC [< 5.7E9/L, ≥ 5.7E9/L], and baseline FBG [< 5.6, ≥ 5.6 mmol/L]). As Fig. 1 indicated, compared with the reference group, the HRs for study participants with MPV level ≥ 9.69 fL were more pronounced in females (HR: 1.56; 95% CI 1.07–2.53), non-current smokers (HR: 1.58; 95% CI 1.17–2.13) and individuals with baseline FPG level < 5.6 mmol/L (HR: 1.95; 95% CI 1.04–3.69) than their counterparts (*P* for interaction was 0.017, 0.013, and 0.021, respectively). In females, further adjustment for number of children, menopausal status, hormone replacement therapy, and contraception status obtained similar results. Females with MPV ≥ 9.80 fL had 92% increased incident risk of DM (95% CI 1.30–2.84) compared with those with MPV < 7.50 fL (*P* for trend = 0.002; see Additional file 2: Table S2). No interaction were found for MPV and age, BMI, current drinker, hypertension, hyperlipidemia, and WBC counts (all *P* for interaction > 0.05; see Additional file 3: Table S3).

## Discussion

In this large cohort study, we found that higher levels of MPV were associated with increased incident risk of diabetes independent of potential confounders. These positive associations were more evident in females, non-current smokers and those with baseline FBG levels less than 5.6 mmol/L.

To our best knowledge, this is the first cohort study to investigate the association between MPV and incident diabetes risk. Accumulating studies have demonstrated that MPV was associated with stroke [22], coronary artery disease [23] and myocardial infarction [24]. A study conducted to investigate the relationship between platelet activity and type 1 diabetes mellitus (T1DM) found that platelet activation did not precede the development of microvascular complications in T1DM [25]. Further investigation in T2DM cases [26] found that individuals with T2DM accompanied by macrovascular disease had higher levels of urine thromboxane metabolite.

Cross-sectional [10] and case–control studies with relative small sample size indicated that person with diabetes or study participants with impaired fasting glucose had higher levels of MPV compared to the healthy controls [15, 27, 28]. A latest meta-analysis enrolling 30 case–control and cross-sectional studies also indicated that MPV was significantly higher among study participants with established diabetes than the healthy controls [14]. However, till now no prospective cohort study was conducted to investigate the associations of MPV levels with incident DM risk. The findings from the present cohort study lend strong support to the positive association between MPV levels and incident DM risk.

Potential mechanisms that underlying this positive association remains unclear. However, several mechanisms might involve in these associations. Firstly, activated platelets could express soluble CD40 ligand (CD40L) [29] and CD40L and its receptor CD40 extensively involved in oxidative stress and inflammatory pathways [30], which may play a role in the development of diabetes [5]. Secondly, higher MPV might partly be due to the regulation of some cytokines, such as IL-6 [31],which could increase the incident diabetes risk by damaging the body’s glucose stability and β-cell function [32]. Notably, the aforementioned associations were more evident in females. Previous studies suggested that gender could influence platelet biology [33]. In the present study, we found a significantly higher level of MPV in females (8.79 fL) than males (8.63 fL), which was consistent with other observations [34]. Oral contraceptives intake and menstruation were strongly associated with higher MPV levels in females [35] which might contribute to sex differences in the MPV level. However, in the present analysis, further adjustment for the number of children, menopausal status, hormone replacement therapy and contraception status did not materially change the positive associations of MPV with incident DM risk in females.

In the present study we found that the positive association of higher MPV and DM incidence was more pronounced in non-current smokers. Smoking was positively associated with increased MPV levels [36], inflammation [37] and immune response [38]. High levels of inflammation in current smokers and relative higher ratio of females in the non-smokers compared to the current smokers (69.2% vs. 6.7%) might contribute to the smoking-MPV interaction on incident DM risk. Similar as smoking, more evident positive association of higher MPV levels and DM incidence was found in those with normal baseline FBG level. Previous studies reported that osmotic swelling due to increased blood glucose could increase MPV levels [27, 28]. Although the underlying pathophysiological mechanisms are unclear, hyperglycemia might cover up the effects of MPV on diabetes among high-risk individuals and leave the detrimental effects robust in relatively healthy population.

The current study has several strengths. Firstly, the prospective cohort study design and relatively large sample size enable us to obtain relatively strong evidence with moderate power. Secondly, the diagnosis of baseline and incident diabetes was based on rigorous criteria, which could reduce the false positive. Thirdly, to the best of our knowledge the present study is the first one to examine the effects of MPV on incident diabetes risk in a middle-aged and older Chinese population, which could provide new insight into diabetes prediction and prevention, especially in middle-aged and older population. Fourthly, in the Cox proportional hazards regression models we have adjusted for multiple confounders including baseline hypertension and hyperlipidemia; thus to some extent the bias of these potential confounders could be minimized. Finally, after exclusion of incident DM diagnosed during the first 2 years of follow-up the positive association was even stronger, indicating the robustness of the positive association.

### Limitations

Some limitations also need to be noted in the present study. Firstly, in the present study MPV was measured only once and might not represent the real MPV levels over time. Secondly, Diaz et al. [39] reported that a progressive increase in MPV with storage in EDTA measured at different time intervals up to 24 h. In the present study blood samples were stored in EDTA anticoagulation tube. However, MPV levels were measured immediately once blood samples were collected. Therefore, the influence from EDTA might be reduced to a small extent. Thirdly, we did not collect information on atrial fibrillation, obstructive sleep apnea, and seasonal changes which is reported to be associated with the MPV levels [40–42]. Missing data on these factors could inevitably lead to potential bias in the evaluation of the HRs for DM. Fourthly, all the participants included in the present study were middle-aged and older Chinese, therefore the findings might not be generalized to younger population or other ethnicities.

## Conclusions

In conclusion, the current findings indicate that MPV, a low-cost, widely available and noninvasive marker, might be a potential risk factor of diabetes in middle-aged and older population. These findings need to be verified in other population with long follow-up period.

## Additional files

**Additional file 1: Table S1.** Baseline characteristics of the study population in women and men.

**Additional file 2: Table S2.** Multivariate adjusted hazard ratios with 95% CI for incident T2DM in women and men.

**Additional file 3: Table S3.** Hazard ratios of T2DM incidence for MPV stratified by characteristics relevant to MPV.

## Abbreviations

DM: diabetes mellitus
MPV: mean platelet volume
T2DM: type 2 diabetes mellitus
T1DM: type 1 diabetes mellitus
WBC: white blood counts
PLT: platelet counts
FBG: fasting blood glucose
HDL-C: high-density lipoprotein cholesterol
LDL-C: low-density lipoprotein cholesterol
CRP: C-reactive protein
TNF-α: tumor necrosis factor-α
IL-6: interleukin-6
CD40L: CD40 ligand
MI: myocardial infarction
CHD: coronary heart diseases
BMI: body mass index
HR: hazard ratio
CI: confidence interval
DMC: Dongfeng Motor Corporation
