# Supplementary material for: Association of mean platelet volume with incident type 2 diabetes mellitus risk: the Dongfeng–Tongji cohort study
Source: Diabetol Metab Syndr. 2018 Apr 10;10:29. doi: 10.1186/s13098-018-0333-6 (PMC5894209; doi:10.1186/s13098-018-0333-6)
Supplement: Supplementary file 1 — Additional file 1: Table S1. Baseline characteristics of the study population in women and men. [file 13098_2018_333_MOESM1_ESM.pdf]

Table S1 Baseline characteristics of the study population in women and men

	Female (n=8029)					Males (n=5980)				
	MPV(fL)					MPV(fL)				
	Q1	Q2	Q3	Q4		Q1	Q2	Q3	Q4	
**Variables**	<7.50	7.50-8.50	8.50-9.80	≥9.80	***P* value**	<7.40	7.40-8.33	8.33-9.52	≥9.52	***P* value**
**Participants**	2037	1980	2036	1976		1501	1498	1493	1488	
**Age, years**	59.57(7.10)	59.80(7.61)	60.12(7.77)	59.75(7.81)	0.376	65.71(6.70)	65.76(6.87)	65.21(6.74)	65.26(6.78)	0.04
**Education, %**					0.593					0.18
**Primary or below**	27.5	27.4	26.1	28.9		30.0	29.9	28.0	31.9	
**Junior high school**	37.6	38.1	39.0	36.4		35.8	34.4	34.8	35.2	
**High school**	28.6	27.9	28.0	27.6		22	21.8	23.0	18.9	
**College or above**	6.2	6.6	7.0	7.1		12.2	13.9	14.2	14.1	
**Body mass index, kg/m^2**	24.37(3.62)	24.11(3.39)	24.00(3.31)	23.97(3.31)	0.008	24.11(3.28)	24.07(3.29)	24.10(3.07)	24.14(3.07)	0.976
**Smoking (yes, %)**	2.4	2.0	2.5	2.0	0.645	41.6	43.8	41.2	40.0	0.211
**Drinking (yes, %)**	7.5	6.6	6.7	7.2	0.584	44.0	43.8	44.7	43.5	0.925
**Physical activity (yes, %)**	87.9	87.2	88.5	88.8	0.411	88.6	87.7	90.5	91.8	0.001
**Family history of diabetes (yes, %)**	6.0	5.9	5.6	4.4	0.056	1.8	1.8	1.7	1.5	0.934
**Hypertension (yes, %)**	24.5	22.9	22.6	23.2	0.791	28.5	24.6	27.6	26.2	0.093
**Hyperlipidemia (yes, %)**	12.3	13.3	13.6	13.2	0.890	13.5	15.0	15.5	13.0	0.217
**Incident diabetes (yes, %)**	7.2	7.1	7.5	6.9	0.147	6.0	6.7	7.9	7.5	0.365
**Menopause (yes, %)**	10.5	12.5	10.6	12.3	0.085	-	-	-	-	-
**Systolic blood pressure (mmHg)**	126.27(18.26)	125.58(18.58)	124.55(18.11)	126.36(18.87)	0.006	130.34(18.62)	129.24(17.96)	129.67(18.23)	128.70(18.24)	0.093
**Diastolic blood pressure (mmHg)**	78.29(10.44)	76.74(10.79)	75.88(10.44)	77.01(10.68)	<0.001	79.36(11.27)	77.89(10.66)	77.77(11.14)	77.25(11.08)	<0.001

**Total cholesterol (mmol/L)**	5.32(0.89)	5.34(0.96)	5.33(0.98)	5.25(0.92)	0.042	4.99(0.86)	4.98(0.86)	4.96(0.89)	4.89(0.88)	0.007
**Triglycerides (mmol/L)**	1.38(0.79)	1.37(1.05)	1.37(0.99)	1.41(0.92)	0.534	1.32(0.93)	1.28(0.80)	1.31(0.89)	1.29(0.79)	0.693
**HDL-cholesterol (mmol/L)**	1.45(0.39)	1.45(0.37)	1.54(0.40)	1.58(0.46)	<0.001	1.42(0.38)	1.34(0.35)	1.39(0.40)	1.46(0.43)	<0.001
**LDL-cholesterol (mmol/L)**	3.11(0.78)	3.17(0.81)	3.06(0.77)	2.96(0.77)	<0.001	2.93(0.80)	3.01(0.76)	2.95(1.07)	2.84(0.76)	<0.001
**Fasting blood glucose (mmol/L)**	5.48(0.54)	5.49(0.52)	5.49(0.57)	5.43(0.61)	0.064	5.58(0.55)	5.62(0.56)	5.59(0.59)	5.47(0.66)	<0.001
**Mean platelet volume, fL**	6.81(0.56)	8.03(0.29)	9.11(0.37)	11.28(1.46)	<0.001	6.75(0.53)	7.88(0.26)	8.89(0.35)	11.02(1.52)	<0.001
**Platelet counts, 10E9/L**	209.59(55.52)	203.86(53.10)	197.43(54.16)	176.87(50.30)	<0.001	189.63(56.02)	189.29(51.45)	180.85(51.17)	164.45(51.25)	<0.001
**White blood cell count, 10E9/L**	5.43(1.49)	5.57(1.39)	5.80(1.48)	5.82(1.62)	0.001	5.99(2.30)	6.21(1.64)	6.27(1.55)	6.32(1.58)	<0.001

Continuous variables were presented as mean (SD). Dichotomous variables were presented as n (%).
