# Supplementary material for: Association of mean platelet volume with incident type 2 diabetes mellitus risk: the Dongfeng–Tongji cohort study
Source: Diabetol Metab Syndr. 2018 Apr 10;10:29. doi: 10.1186/s13098-018-0333-6 (PMC5894209; doi:10.1186/s13098-018-0333-6)
Supplement: Supplementary file 2 — Additional file 2: Table S2. Multivariate adjusted hazard ratios with 95% CI for incident T2DM in women and men. [file 13098_2018_333_MOESM2_ESM.pdf]

Table S2 Multivariate adjusted hazard ratios with 95% CI for incident T2DM in women and men

		Female (n=8029)*		*P* trend&			Males (n=5980)#		*P* trend&
**MPV quartile**	**MPV(fL)**	**Case/ Person-years**	**HR(95%CI)**		**MPV quartile**	**MPV(fL)**	**Case/ Person-years**	**HR(95%CI)**	
				0.002					0.933
Q1	<7.50	168/8936.45	Ref		Q1	<7.40	90/6507.48	Ref	
Q2	7.50-8.50	147/8604.87	1.44(1.03,2.00)		Q2	7.40-8.33	101/6342.80	1.26(0.89,1.79)	
Q3	8.50-9.80	172/8917.30	1.56(1.09,2.22)		Q3	8.33-9.52	118/6430.09	0.85(0.60,1.21)	
Q4	≥9.80	144/8584.36	1.92(1.30,2.84)		Q4	≥9.52	111/6438.39	1.09(0.75,1.59)	

*: adjusted for age, BMI, smoking status, drinking status, education, physical activity, hypertension, hyperlipidemia, family history of DM, number of children, menopausal status, hormone replacement therapy, contraception status, examination center ,WBC, and PLT.

#: adjusted for age, BMI, smoking status, drinking status, education, physical activity, hypertension, hyperlipidemia, family history of DM, examination center, WBC, and PLT.

&: *P* value when assigning the median value to each quartile and entered this as a continuous variable in the models.
