# Supplementary material for: Association of mean platelet volume with incident type 2 diabetes mellitus risk: the Dongfeng–Tongji cohort study
Source: Diabetol Metab Syndr. 2018 Apr 10;10:29. doi: 10.1186/s13098-018-0333-6 (PMC5894209; doi:10.1186/s13098-018-0333-6)
Supplement: Supplementary file 3 — Additional file 3: Table S3. Hazard ratios of T2DM incidence for MPV stratified by characteristics relevant to MPV. [file 13098_2018_333_MOESM3_ESM.pdf]

Table S3 Hazard ratios of T2DM incidence for MPV stratified by characteristics relevant to MPV

	n#	MPV quartile				*P* for trend *	*P* for interaction
		Q1	Q2	Q3	Q4		
Age, years							0.073
<60	5075	Ref	1.53(0.98,2.83)	1.66(1.08,2.56)	2.07(1.27,3.37)	0.006	
≥60	8934	Ref	1.35(1.03,1.77)	0.93(0.69,1.24)	1.11(0.81,1.53)	0.87	
Gender							0.017
Male	5980	Ref	1.40(0.99,1.97)	0.84(0.59,1.18)	1.25(0.85,1.84)	0.71	
Female	8025	Ref	1.40(1.02,1.93)	1.47(1.05,2.05)	1.56(1.07,2.53)	0.05	
BMI, kg/m^2							0.429
<25	8673	Ref	1.64(1.15,2.33)	1.41(0.98,2.05)	1.68(1.11,2.54)	0.45	
≥25	5158	Ref	1.22(0.90,1.65)	1.00(0.73,1.37)	1.23(0.87,1.75)	0.32	
Current Smoking							0.013
No	11231	Ref	1.40(1.08,1.82)	1.31(1.00,1.71)	1.58(1.17,2.13)	0.01	
Yes	2670	Ref	1.67(0.94,2.97)	0.68(0.40,1.16)	0.95(0.52,1.71)	0.23	
Current Drinking							0.428
No	10800	Ref	1.16(0.91,1.48)	0.98(0.77,1.25)	1.19(0.93,1.51)	0.33	
Yes	3192	Ref	1.69(1.09,2.63)	1.25(0.79,1.96)	1.41(0.89,2.23)	0.43	
Hypertension							0.972
No	10520	Ref	1.49(1.11,1.99)	1.20(0.89,1.62)	1.49(1.06,2.08)	0.29	
Yes	3469	Ref	1/28(0.87,1.89)	1.10(0.74,1.65)	1.37(0.88,2.16)	0.23	
Hyperlipdemia							0.670
No	12079	Ref	1.39(1.07,1.82)	1.13(0.87,1.47)	1.29(0.97,1.74)	0.42	
Yes	1901	Ref	1.67(0.97,2.86)	1.29(0.72,2.34)	2.09(1.08,4.03)	0.19	
WBC counts							0.887

< median (5.7E9/L)	6445	Ref	1.27(0.89,1.79)	1.14(0.78,1.66)	1.52(1.01,2.29)	0.07	
≥median (5.7E9/L)	6254	Ref	1.53(1.11,2.09)	1.18(0.86,1.61)	1.37(0.97,1.94)	0.35	
FBG, mmol/L							0.021
<5.6	7741	Ref	1.56(0.96,2.56)	1.38(0.79,2.43)	1.95(1.04,3.69)	0.06	
≥5.6	6225	Ref	1.28(0.98,1.67)	1.10(0.84,1.44)	1.25(0.93,1.67)	0.31	

Model 3 in Table 2 was used in this analysis.

#: Some characteristics have missing values hence not the same total n for each stratification characteristics.

*: P value when we assigned the median value to each quartile and entered this as a continuous variable in the model;
